# Data warehouse and medical research

**DOI:** 10.31744/einstein_journal/2022ED6324

**Published:** 2022-02-23

**Authors:** Thiago Gonçalves dos Santos Martins, Flavia de Souza Rangel

**Affiliations:** 1 Universidade de Coimbra Coimbra Portugal Universidade de Coimbra, Coimbra, Portugal.; 2 Universidade Estácio de Sá Rio de Janeiro RJ Brazil Universidade Estácio de Sá, Rio de Janeiro, RJ, Brazil.

In the last 2 years, about 90% of the world’s data has been generated. The great increase in data production in medicine has often exceeded the capacity of processing them. This large production has allowed the development of multicenter epidemiological studies with large numbers of participants, and large amounts of data generated. This facilitates data generation about the development and popularization of electronic medical records at hospitals and medical research centers around the globe.^(1)^

The development of new technologies for the interpretation and selection of these data has been of great importance in medicine. In addition, this allows the advancement of research with a larger number of participants aligned to a lower cost and shorter time invested.^(2)^ Among the technologies used for this purpose is the use of data warehouse.

This data storage program enables the analysis of large amounts of information in a short period of time. The program makes it possible to store and organize the collected data and to search for large amounts of data (“data mining”). The results obtained by data mining enable the improvement of classifications and associations of the analyzed information.

The studies performed with this large amount of data provide better clinical decision making and statistically significant results in less time compared with traditional epidemiological studies. For this reason, the data warehouse is useful for processing large amounts of medical data, which can be used to support decision making based on valid and reliable information. In addition, these data can be stored for months or years.^(3,4)^

The use of the data warehouse allows real-time data analysis and provides a better development of public policies in the area of healthcare. In the area of public management, the examination of big data has focused on improving the quality of services provided to individuals, and the optimization of public resources. For technologies to work, they need to be adapted to the context and culture of the environment in which they will be applied. To do this, we must consider the particularities of each location and action to encompass digital accessibility, digital inclusion, scalability, and technological sustainability. Furthermore, the issue of digital accessibility and inclusion is the major challenge to ensure that citizens, broadly speaking, to have access to all services provided by the government.

Considering this issue, we must pay attention to the protection of research data, according to the laws of the place of the study. In Brazil, the right to the protection of personal data in digital media is not yet expressly included in the Constitution as a fundamental right. However, there is an ongoing constitutional amendment proposal number 17/2019 that aims to insert Article 5, XII-A, CRFB/88, to ensure the fundamental right to the protection of personal data as well as safety in digital media. Despite the existence of Articles 5, X and XII of the Brazilian Constitution, the guarantee of data protection is still insufficient in the current information age.^(5)^

In this context, the Federal Supreme Court recently recognized the data protection as an autonomous fundamental right, in a paradigmatic case regarding the request for sharing citizens’ personal data by telephone companies with the *Instituto Brasileiro de Geografia e Estatística* (IBGE) during the coronavirus pandemic in 2020. The decision was historical, and its degree of importance is compared by experts to the German Constitutional Court in 1983, which brought to the country the concept of informational self-determination.^(6,7)^

At the infra-constitutional level, the General Data Protection Law - LGPG *(Lei Geral de Proteção de Dados Pessoais* - law 13.709 of August 14, 2018) after a 2-year period and numerous discussions is currently in force in Brazil.^(8)^ However, there are exceptions such as the embryonic implementation of the National Data Protection Authority, whose amendment of the LGPD, through the law 13.853/2019, provided for its creation with the legal nature of a public body, and its structure is regulated by Ordinary 10.474 of August 26, 2020.^(9)^ This law recently entry into force after the publication of the appointment of the Chief Executive Officer of the Board of Directors of the National Data Protection Authority (Federal Official Gazette (*Diário Oficial da União*), November 6, 2020), as expressed in Article 6 of the mentioned ordinary. However, in this regard, there are still several difficulties to be faced in this new reality.

In European countries, such as Portugal, the fundamental right to data protection is not new, being a theme pertaining to its Constitution and present in the Fundamental Rights of the European Union.^(10)^Of note is that, in a context of the information age, the General Data Protection Regulation (GDPR; regulation 2016/679 of the European Parliament and Council) was issued on April 27, 2016, and became effective from May 25, 2018. It is important to highlight that the Brazilian LGPD was greatly inspired by the European GDPR, although there are divergences. Portugal subsequently edited the law 58/2019,^(11)^replacing it with the law 67/98 on the Personal Data Protection Law, which is currently in force.^(12)^

The creation of artificial intelligence algorithms has allowed the development of data warehouses with autonomy for data extraction and analysis. This automatization can reduce the human errors often found in the manual documentation of data, and this has been an alternative for studies with larger amounts of collected information. The data warehouse can extract data from the cloud, reduce the costs and complexity of the process, and facilitate the decision making in real time,^(13)^ which would compensate the high investments to implement these programs.

Finally, there is need to highlight that data warehouse is currently unable to conduct adequate analysis of unstructured data, such as images and graphs. In addition, possible errors in the documentation can hinder the final analysis of the results. Therefore, it is important to involve the physician during the whole process of making and running the program in an attempt to ensure the quality of the data generated, and avoid that the big data analyzed produces a “big noise”, and may generate false correlations between the investigated data.
